# Hippocampal Metabolomics Reveal the Mechanism of α-Conotoxin [S9K]TxID Attenuating Nicotine Addiction

**DOI:** 10.3390/md24010043

**Published:** 2026-01-15

**Authors:** Meiting Wang, Weifeng Xu, Huanbai Wang, Cheng Cui, Rongyan He, Xiaodan Li, Jinpeng Yu, J. Michael McIntosh, Dongting Zhangsun, Sulan Luo

**Affiliations:** 1 Guangxi Key Laboratory of Special Biomedicine, School of Medicine, College of Life Science and Technology, Guangxi University, Nanning 530004, China; wmttymt@163.com (M.W.); 19071010110011@hainanu.edu.cn (W.X.); hbwang93@163.com (H.W.); cuicuicuicheng@163.com (C.C.); herongyan@gxu.edu.cn (R.H.); yujinpeng@gxu.edu.cn (J.Y.); 2 Key Laboratory of Tropical Biological Resources of Ministry of Education, Hainan University, Haikou 570228, China; 993586@hainanu.edu.cn; 3 Departments of Biology and Psychiatry, University of Utah, Salt Lake City, UT 84112, USA; mcintosh.mike@gmail.com; 4 George E. Wahlen Veterans Affairs Medical Center, Salt Lake City, UT 84108, USA

**Keywords:** α3β4 nAChRs, α-conotoxin [S9K]TxID, nicotine addiction, CPP, metabolomics

## Abstract

Nicotine is the main substance responsible for the development of tobacco addiction. The α3β4 nicotinic acetylcholine receptors (nAChRs) are a potential key target for mitigating nicotine reward. Preliminary studies in our laboratory suggest that α-conotoxin [S9K]TxID serves as a selective and potent antagonist targeting α3β4 nAChRs, which may be beneficial in addressing nicotine addiction. However, the mechanisms of [S9K]TxID treatment in nicotine addiction are still to be determined. This study aimed to identify the differential metabolic profiles of [S9K]TxID treatment in nicotine addiction using an untargeted metabolomic profiling method. As demonstrated by behavioral experiments, [S9K]TxID effectively attenuated nicotine-induced conditioned place preference (CPP) expression without exerting inhibitory effects on the central nervous system (CNS). The results of untargeted metabolomics revealed that eight metabolites were significantly altered after [S9K]TxID treatment, particularly phenylalanine. [S9K]TxID also attenuated nicotine-induced metabolic disorders by regulating phenylalanine, tyrosine and tryptophan biosynthesis. In conclusion, our findings suggest that [S9K]TxID could be a potential therapeutic compound for nicotine addiction.

## 1. Introduction

The utilization of tobacco presents a considerable challenge to public health on a global scale. Data from the World Health Organization (WHO) indicate that tobacco consumption is responsible for approximately eight million deaths annually worldwide, with approximately seven million of these fatalities linked to individuals who are current smokers [1]. China holds the status of being the foremost producer and consumer of tobacco globally. The impact of smoking on public health is significant, resulting in a considerable health burden. A survey has shown that the prevalence of smoking in China has remained at a high level since 2003, with a significant increase in the prevalence of smoking among adolescents and young women. Smoking, particularly early and long-term smoking, is strongly linked to an elevated risk of chronic diseases. According to the report, if the prevalence of smoking continues to rise, it is expected that the number of deaths due to smoking will reach two million per year by 2030 [2]. A variety of smoking cessation therapies are currently available, including nicotine replacement therapy (NRT) [3], the antidepressant bupropion [4], and the partial nicotine agonist varenicline [5]. However, while NRT reduces the patient’s cravings for nicotine, it still maintains the physiological and behavioral dependence on nicotine. Bupropion and varenicline may cause serious unwanted effects (e.g., insomnia, gastrointestinal problems, or suicidal ideation). Therefore, the study of anti-addiction drugs is of greater clinical significance.

Nicotine has been identified as the principal addictive substance in tobacco smoke and acts on nAChRs in both the central and peripheral nervous systems [6,7]. Multiple investigations indicate that the α3β4 nAChR subtype is associated with a wide variety of drugs of abuse in addition to nicotine addiction [8,9]. These α3β4 nAChRs are distributed throughout the brain, with high concentrations in the medial habenula (MHb) and interpeduncular nucleus (IPN) [10,11]. Furthermore, their presence extends to additional regions, including the ventral tegmental area, dorsolateral tegmentum, and basolateral amygdala [12,13,14,15]. The MHb-IPN pathway serves as a recognized target for nicotine, modulated by the α3β4 nAChRs, either directly or indirectly interrelated with the dopaminergic mesolimbic system [16]. Glick et al. demonstrated that the α3β4 nAChRs antagonist 18-methoxycoronidine (18-MC) reduces the self-administration of nicotine [17,18]. The selective antagonist AT-1001 of the α3β4 nAChRs has the ability to inhibit intravenous self-administration of nicotine in a dose-dependent manner following systemic injection in rats. In addition, AT-1001 has been demonstrated to inhibit behavioral sensitization in mice [19,20,21]. Overall, α3β4 nAChRs are important targets for addiction drugs, and the advancement of antagonists for these receptors may serve as a promising approach for addressing addiction treatment.

α-Conotoxins, as antagonists of nAChRs, can be used as a drug lead or molecular probe against diseases related to nAChR function [22]. α-conotoxin AuIB, the first α3β4 nAChR-selective antagonist, was identified from *Conus aulicus*, but its low potency has limited its use in functional studies [23,24]. Our laboratory identified a novel α-conotoxin TxID from *Conus textile*. This compound demonstrates a significant ability to block the rat α3β4 nAChRs while concurrently inhibiting the α6/α3β4 nAChR [25]. We subsequently developed a highly selective α3β4 nAChRs antagonist [S9K]TxID by a single amino acid substitution strategy, which targets α3β4 nAChRs with an IC_50_ of 6.9 nM for the rat receptor [26]. To date, it is the most selective α3β4 nAChRs antagonist that has been identified. In previous animal behavioral tests, we found that TxID and [S9K]TxID inhibited nicotine-induced CPP expression and relapse while having no significant effect on acute nicotine exposure [27]. However, these studies primarily focused on the therapeutic effects of [S9K]TxID on nicotine addiction, without delving into its underlying mechanisms. To address this gap, we aimed to explore whether [S9K]TxID treatment of nicotine addiction was related to certain endogenous metabolites within the brain. Therefore, our study employed metabolomics to analyze the changes of metabolites in the hippocampal brain region after [S9K]TxID treatment in mice.

In this study, the α3β4 nAChRs selective antagonist [S9K]TxID was employed to examine the pharmacodynamic properties of α3β4 nAChRs in a mouse model of nicotine-induced CPP. Alteration of hippocampal tissue metabolites by [S9K]TxID was analyzed using metabolomics. This study provides a basis for further research of its mechanism of action.

## 2. Results

### 2.1. Mouse Behaviors in Open Field Test (OFT) and Elevated Plus Maze Test (ELPM) After [S9K]TxID Treatment

**Figure 1:**
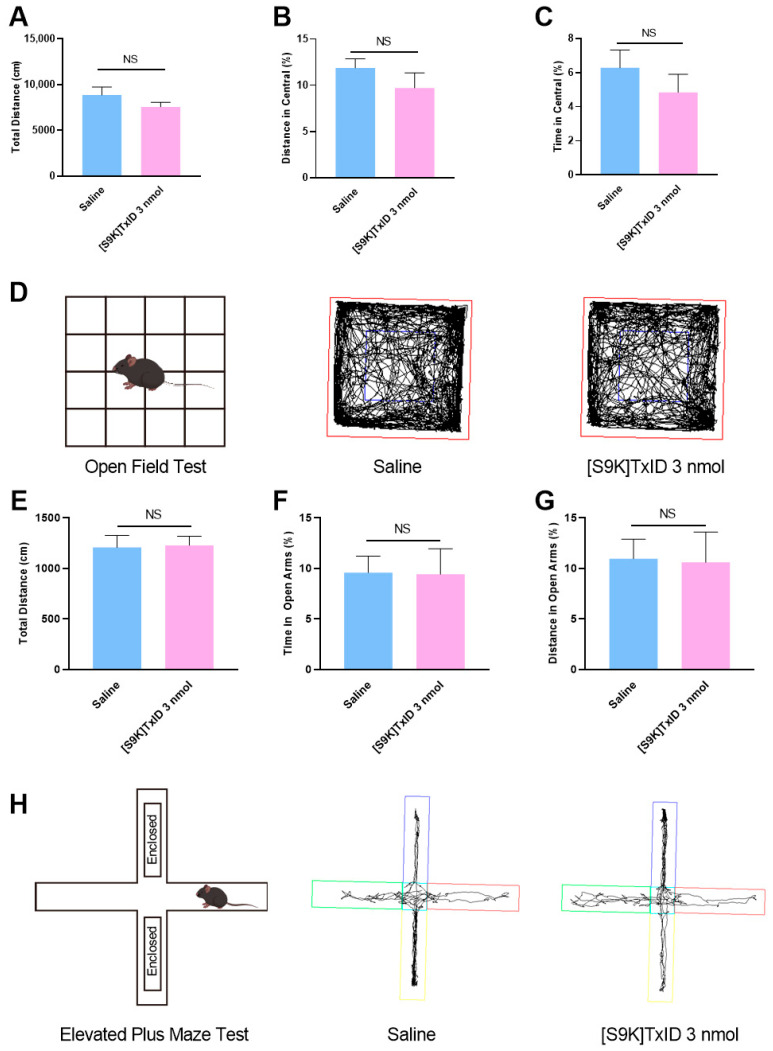
[S9K]TxID treatment does not induce mouse anxiety-related behaviors. (**A**–**C**) [S9K]TxID treatment did not alter Total distance, Distance in Central (%) or Time in Central (%); (**D**) Mouse movement trajectory; (**E**–**G**) [S9K]TxID did not induce anxiety as indicated by no alteration in Total Distance; Time in Open Arms (%); Distance in Open Arms (%); (**H**) Mouse movement trajectory in elevated plus maze. NS: not significant (Mean ± SEM, *n* = 8).

A series of behavioral tests was conducted to assess the possibility that [S9K]TxID treatment was affecting basal motor activity and other behaviors that might impede CPP formation. Mice were treated with [S9K]TxID three nmol or saline (i.c.v.) 90 min prior to the behavioral test. In OFT, there was no significant difference between the saline group and the [S9K]TxID three nmol group in terms of total distance (Figure 1A), distance in central (Figure 1B), and time in central (Figure 1C). In ELPM, there was no significant difference between the saline group and the [S9K]TxID three nmol group in terms of total distance (Figure 1E), time in open arms (Figure 1F), and distance in open arms (Figure 1G).

### 2.2. [S9K]TxID Attenuates Expression of Nicotine-Induced CPP in Mice

**Figure 2:**
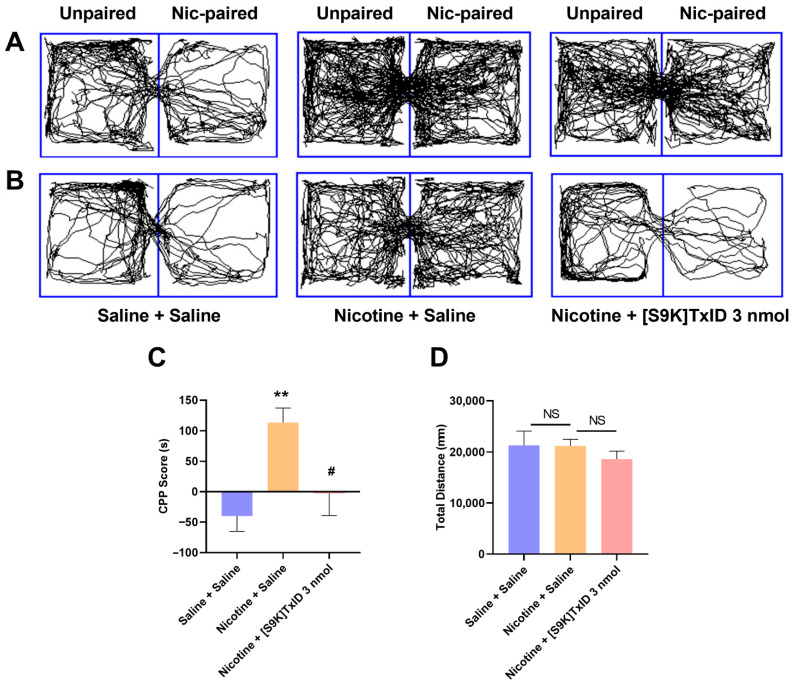
[S9K]TxID attenuates expression of nicotine-induced CPP in mice. (**A**) The trajectory of the mice in the CPP box prior to the administration of the antagonist [S9K]TxID; (**B**) The trajectory of the mice in the CPP box after the administration of the antagonist [S9K]TxID; (**C**) CPP scores in each group after injection of saline or [S9K]TxID; (**D**) The total distance during the 15 min test phase. ** *p* < 0.01 compared with Saline + Saline group; ^#^ *p* < 0.05 compared with the Nicotine + Saline group. NS: not significant (Mean ± SEM, *n* = 10).

After a 5-day nicotine training regimen at a dosage of 0.5 mg/kg, the time spent in drug-paired compartments by mice administered nicotine was significantly different than the control group (*p* < 0.01) (Table 1). The trajectory of the mice of each group in the CPP box, prior to the administration of the antagonist [S9K]TxID, is illustrated in Figure 2A. As can be seen from the figure, the Nicotine + Saline and Nicotine + [S9K]TxID groups showed increased trajectories in the drug-paired compartments compared to the Saline + Saline group. [S9K]TxID treatment attenuates expression of nicotine-induced CPP in mice (Table 2). Following the administration of the antagonist [S9K]TxID, the trajectories of the mice of each group in the CPP box are illustrated in Figure 2B. Compared with the Saline + Saline group, the CPP scores were significantly higher in the Nicotine + Saline group (*p* < 0.01). Mice treated with [S9K]TxID demonstrated significantly reduced CPP scores than those in the Nicotine + Saline group (*p* < 0.05) (Figure 2C). As can be seen from the figure, in contrast to the Nicotine + Saline group, the Nicotine + [S9K]TxID group displayed a reduced trajectory in the drug-paired compartments. No significant differences were observed in the total distance traveled by each group (Figure 2D).

### 2.3. Metabolomic Profiles

#### 2.3.1. Quality Evaluation of Metabolomics Data

**Figure 3:**
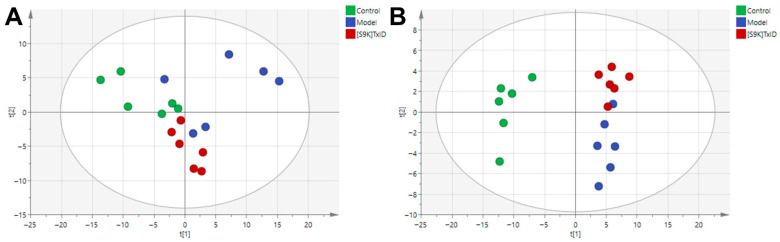
Plots of PCA scores for Control, Model, and [S9K]TxID groups in positive (**A**) and negative (**B**) ion modes. Each point in the PCA score plot represents a sample. In the plots of PCA scores, green points represent the Control group, blue points represent the Model group, and red points represent the [S9K]TxID group.

To explore the effect of [S9K]TxID on metabolic fingerprints, we performed an untargeted metabolomics analysis on the hippocampus in the Control group (Saline + Saline), Model group (Nicotine + Saline), and [S9K]TxID group (Nicotine + [S9K]TxID 3 nmol). The typical total ion chromatograms (TICs) of mouse hippocampus samples are presented in Figures S1 and S2. The LC-MS raw data were imported into Compound Discoverer software (CD version 3.3) for peak identification, normalization, and alignment. The resultant data was imported to SIMCA 14.1 software for multivariate statistical analyses, including principal component analysis (PCA) and orthogonal partial least squares discriminant analysis (OPLS-DA). PCA is a data analysis technique that employs multidimensional space to visualize the differences between samples [28]. Quality control (QC) samples were evaluated for stability and reproducibility of metabolomics. QC samples were evaluated for stability and reproducibility of metabolomics. The PCA plots revealed that the QC samples exhibited good clustering in positive and negative ion modes, thereby substantiating the reliability of the experimental conditions (Figure S3). The distribution of the Control group, Model group, and [S9K]TxID group in plots of PCA scores tended to separate, indicating that nicotine-addicted mice had significantly altered hippocampus metabolite profiles. The metabolic profiles of the [S9K]TxID group were closer to the control group, with an overlap between the Model group, indicating that [S9K]TxID treatment modulated the nicotine-induced dysregulation of metabolism (Figure 3A,B).

#### 2.3.2. [S9K]TxID Modulated Hippocampus Metabolomic Profiling

**Figure 4:**
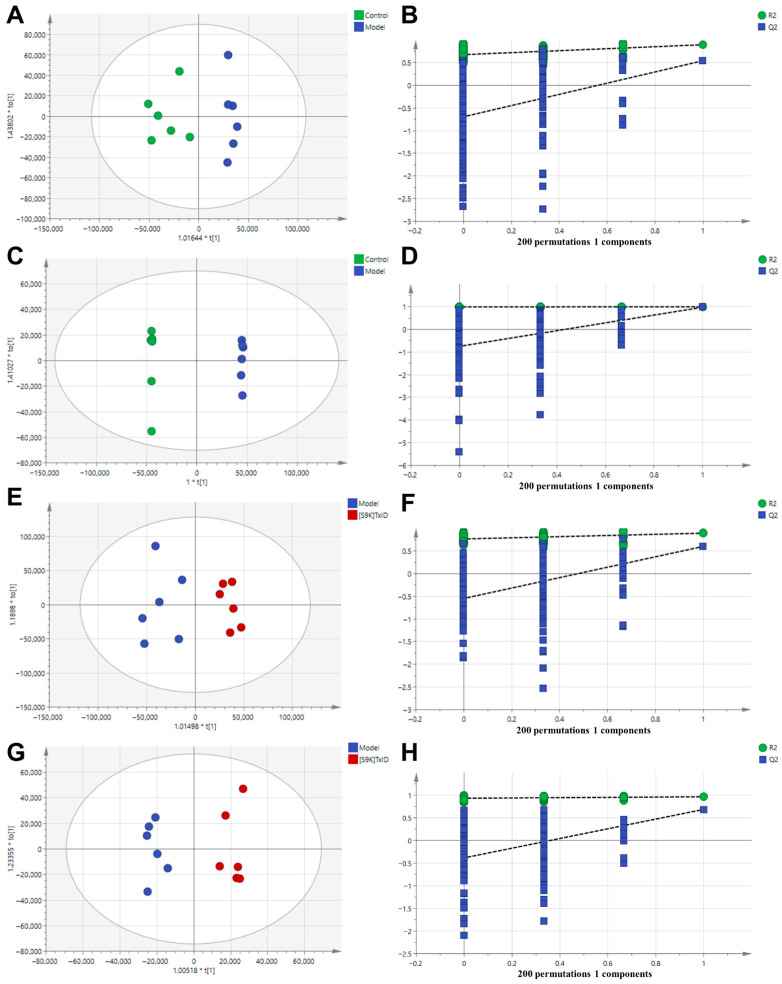
OPLS-DA score plots and permutation tests for hippocampus metabolites in positive and negative ion modes. (**A**,**B**) OPLS-DA score plot and permutation test in positive ion mode between Control group and Model group. (**C**,**D**) OPLS-DA score plot and permutation test in negative ion mode of Control group and Model group. (**E**,**F**) OPLS-DA score plot and permutation test in positive ion mode between Model group and [S9K]TxID group. (**G**,**H**) OPLS-DA score plot and permutation test in negative ion mode between Model group and [S9K]TxID group. In the OPLS-DA score plots, green points represent the Control group, blue points represent the Model group, and red points represent the [S9K]TxID group. R2 is the explained variance, and Q2 is the predictive ability of the model.

To explore the different metabolites in each group, OPLS-DA was employed. OPLS-DA models were performed between Control vs. Model and Model vs. [S9K]TxID, respectively. The Control group was significantly differentiated from the Model group samples, with evaluation indexes R2Y = 0.897, Q2 = 0.544 (positive ion mode), and R2Y = 1, Q2 = 0.979 (negative ion mode) (Figure 4A,C), suggesting that the model is stable and predictable. The Model group was also significantly differentiated from the [S9K]TxID group, with evaluation indexes R2Y = 0.895, Q2 = 0.602 (positive ion mode), and R2Y = 0.963, Q2 = 0.685 (negative ion mode) (Figure 4E,G), suggesting that the model is stable and predictable. The y-axis and the intersection of the blue regression lines of the Q2 points in the permutation plot were negative, showing that all of the OPLS-DA models were not overfitting (Figure 4B,D,F,H).

**Figure 5:**
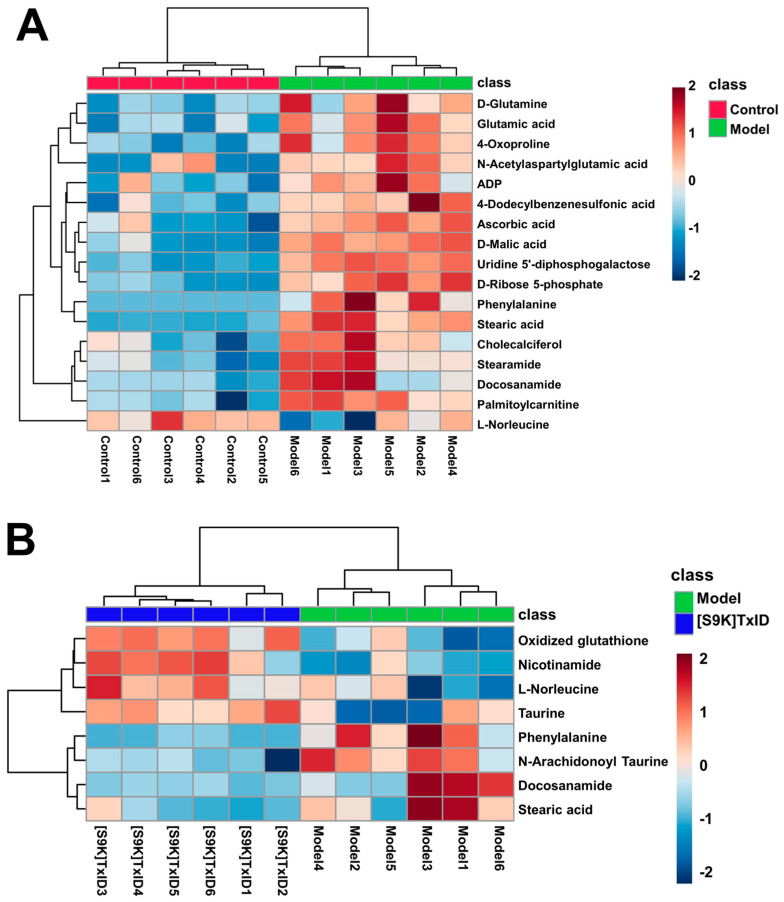
Heatmap of significantly altered metabolites in each group. (**A**) Hierarchical clustering heatmap of the Control group and Model group. (**B**) Hierarchical clustering heatmap of the [S9K]TxID group and Model group.

A total of 88 metabolites were identified by the Human Metabolome Database (HMDB) and PubChem databases (Table S1). The differential metabolites were screened using the variable importance in projection (VIP) > 1 and *p* < 0.05 criteria. The results show that, in contrast to the Control group, there were alterations in 17 metabolites within the Model group; similarly, when contrasting the Model group to the [S9K]TxID group, eight metabolites exhibited changes (Table 3). The ratio values of differential metabolites are provided in the Supplementary Table S2. In order to compare metabolite content variations in different groups, the metabolite content of hippocampal samples was converted into a visualized clustered heatmap using the MetaboAnalyst 6.0 online metabolomics tool. Each column in the heatmap represented a sample, and each square represented the abundance of a differential metabolite in a sample. Blue represents low content, and red represents high content. Large differences between groups and small differences within groups were shown by the clustering analysis of differential metabolites (Figure 5A,B). Specifically, in the Model group, there was an observed upregulation of 16 metabolites (such as 4-dodecylbenzenesulfonic acid and stearic acid), while one metabolite (L-norleucine) exhibited downregulation in comparison to the Control group. In the [S9K]TxID group, there was an observed upregulation of four metabolites (such as taurine and nicotinamide), while four metabolites (including phenylalanine and docosanamide) exhibited downregulation in comparison to the Model group. Following the removal of metabolites with analogous trends in the Model and [S9K]TxID groups, four differential metabolites exhibited a tendency towards the Control group following treatment with [S9K]TxID. The results indicate that [S9K]TxID treatment induced alterations in metabolites within the hippocampus of nicotine-addicted mice.

#### 2.3.3. [S9K]TxID Regulated Metabolomic Pathways in Nicotine-Addicted Mice

**Figure 6:**
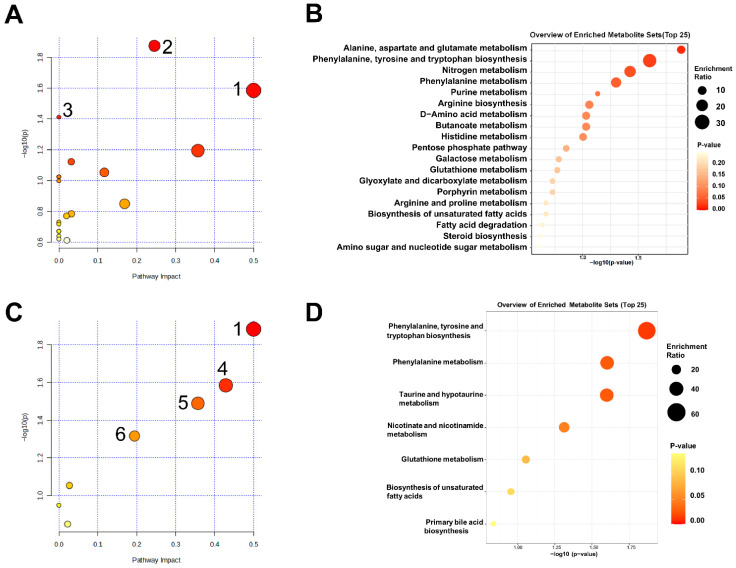
Analysis of metabolic pathways of hippocampal metabolites. (**A**) Metabolic pathway analysis of metabolites in Control and Model groups. (**B**) Enrichment analysis of metabolites in Control and Model groups. (**C**) Metabolic pathway analysis of metabolites in [S9K]TxID group and Model group. (**D**) Enrichment analysis of metabolites in [S9K]TxID group and Model group. (1) Phenylalanine, tyrosine and tryptophan biosynthesis. (2) Alanine, aspartate and glutamate metabolism. (3) Nitrogen metabolism. (4) Taurine and hypotaurine metabolism. (5) Phenylalanine metabolism. (6) Nicotinate and nicotinamide metabolism.

We performed enrichment analysis and pathway analysis of different metabolites using the Kyoto Encyclopedia of Genomes (KEGG) and MetaboAnalyst 6.0 to identify metabolic pathways that may be regulated by [S9K]TxID in nicotine-addicted mice. Figure 6A displays the outcomes of the pathway analysis for the Model and Control groups. The analyses showed that a total of 19 metabolic pathways were enriched, and three metabolic pathways were identified as showing significant differences (*p* < 0.05). The size of each bubble, which stands for a metabolic pathway, is proportionate to the impact of that pathway; the color of the bubbles indicates the significance of each pathway, ranging from highest (red) to lowest (white). These pathways included phenylalanine, tyrosine and tryptophan biosynthesis; alanine, aspartate and glutamate metabolism; as well as nitrogen metabolism. The enrichment analysis was used to support the results above (Figure 6B). Each bubble represents a metabolic pathway, and the *p* value was represented by the circles’ color depth. It can be inferred that nicotine intake may induce alterations in the aforementioned related metabolic pathways.

Four of the seven metabolic pathways linked to the [S9K]TxID therapy of nicotine-addicted mice exhibited significant differences, according to the enrichment and pathway analysis of Figure 6C,D. These pathways included phenylalanine, tyrosine and tryptophan biosynthesis; taurine and hypotaurine metabolism; phenylalanine metabolism; as well as nicotinate and nicotinamide metabolism. After comparing these results with the pathways shared by the Model and Control groups, it can be inferred that the potential metabolic pathway for [S9K]TxID treatment of nicotine addiction is the phenylalanine, tyrosine and tryptophan biosynthesis. It can be inferred that [S9K]TxID mainly plays a role in the treatment of nicotine-addicted mice by regulating amino acid metabolism.

## 3. Discussion

Smoking is a chronic, relapsing disease with significantly increasing incidence and mortality rates worldwide [29]. It is characterized by compulsive smoking and abuse and involves neural circuits for cognitive and emotionally relevant behaviors. These may be due to pathological changes in different brain regions [30]. Tobacco dependence is fundamentally nicotine dependence, with nicotine being the primary psychoactive component in tobacco. Due to the complex mechanisms of nicotine addiction, most patients relapse despite existing treatment options, and clinically effective therapeutic agents remain scarce. Therefore, there is an urgent need to develop highly effective and safe medications for smoking cessation.

The results of our investigation substantiate that [S9K]TxID exhibits a notable therapeutic impact by diminishing nicotine-induced CPP expression, aligning with our earlier research and the findings reported by Jackson et al. [27,31]. Currently, the development of compounds for targeting α3β4* nAChRs has become a major research focus in the field of nicotine addiction. For instance, Glick et al. reported that local injection of the α3β4 nAChR antagonist 18-MC or AuIB reduced nicotine self-administration in rats [17]. Mccallum et al. reported that pretreatment with intra-habenular 18-MC or AuIB inhibited the increases in extracellular dopamine levels in the nucleus accumbens (NAcc) that were caused by nicotine [18]. Toll et al. provided evidence that the antagonist AT-1001 of the α3β4 nAChRs markedly reduced nicotine self-administration behavior in rats [19]. These findings suggest that α3β4 nAChRs could be important targets for nicotine addiction treatment and that α3β4 nAChR antagonists hold promise as potential smoking cessation medications.

We performed a series of behavioral tests after [S9K]TxID treatment. The results showed no significant effects in the OFT and ELPM experiments in either group indicating that [S9K]TxID has no inhibitory effect on the CNS in these tests. The above experiments demonstrated that receiving [S9K]TxID treatment significantly reduced CPP scores in mice. However, nicotine addiction affects changes in metabolites in brain regions. Therefore, evaluating only the efficacy of [S9K]TxID in nicotine-addicted mice is usually insufficient, and thus, we explored the differential metabolic profiles of [S9K]TxID treatment in nicotine addiction by studying hippocampal metabolites.

Untargeted metabolomics serves as a widely employed technique for identifying potential small-molecule metabolites that are influenced by disease states or pharmacological interventions. This study involved the screening of differential metabolites linked to nicotine addiction and modulated by [S9K]TxID through untargeted metabolomics analysis. In comparison to the Model group, eight metabolites changed in the [S9K]TxID group. The findings from the KEGG enrichment analysis indicated that there was a predominant enrichment in seven metabolic pathways. By comprehensively analyzing these differential metabolites, we found that the most relevant pathways are the phenylalanine, tyrosine and tryptophan biosynthesis. Following the removal of metabolites with analogous trends in the model and [S9K]TxID groups, four different metabolites were reversed after [S9K]TxID treatment.

[S9K]TxID regulates the phenylalanine, tyrosine and tryptophan biosynthesis. Phenylalanine holds substantial significance in relation to nicotine addiction. Nicotine functions as a crucial component in the realm of tobacco dependence and demonstrates a wide range of pharmacological properties within the central and peripheral nervous systems. Studies have shown that nicotine induces an increase in the release of glutamate, dopamine, serotonin, and acetylcholine in the hippocampus, cerebellum, nucleus ambiguus, and striatum [32,33,34]. Phenylalanine, as a precursor substance to dopamine, norepinephrine, and epinephrine, further affects the levels of neurotransmitters, which are closely linked to the stages of addiction onset, development, and withdrawal. The role of phenylalanine in neuropathology and as a parameter for monitoring brain monoamine function has previously been studied [35]. In the present study, phenylalanine was upregulated in the hippocampal tissue of the Model group, which could be reversed by [S9K]TxID intervention, suggesting that [S9K]TxID could achieve a therapeutic effect by regulating phenylalanine, tyrosine and tryptophan biosynthesis.

Taurine is an inhibitory neurotransmitter with neuroprotective and neurotrophic properties that are essential for the maintenance of neuronal functions [36]. It has been demonstrated that taurine protects cells and tissues from inflammation and oxidative stress [37,38]. Based on studies, taurine injections administered intraperitoneally significantly reduced the extent of bladder and kidney damage and dysfunction brought on by chronic nicotine administration [39]. Our results show, in comparison to the model group, taurine levels in hippocampal tissue increased to normal levels after [S9K]TxID treatment. Thus, [S9K]TxID may treat nicotine addiction by reducing nicotine-induced oxidative stress and neuroinflammation through increasing taurine levels.

Nicotinamide represents the amide derivative of vitamin B3, commonly known as niacin. Nicotinamide serves as a precursor to the coenzymes nicotinamide adenine dinucleotide (NAD^+^) and nicotinamide adenine dinucleotide phosphate (NADP^+^). These coenzymes are integral to the maintenance of normal cellular functions, contributing to energy metabolism, DNA repair, the aging process, and responses to oxidative stress [40]. Nicotinamide serves as a crucial precursor for NAD^+^/NADH, which is essential for DNA biosynthesis. The tricarboxylic acid (TCA) cycle represents a crucial metabolic pathway found in aerobic organisms. Nicotinamide plays a significant role in energy metabolism within this cycle, employing NAD^+^ in the mitochondrial respiratory electron transport chain to produce ATP as well as to facilitate DNA synthesis and repair processes [41,42,43]. The process of addiction is one that requires a consumption of energy. Studies have shown that in nicotine-induced CPP mice, NAD content is significantly reduced in the prefrontal cortex of the brain, causing energy metabolism disorders [44]. In the present study, [S9K]TxID upregulated nicotinamide content and thus was linked to regulation of dysregulated energy metabolism in nicotine-addicted mice.

Since our study confirmed that i.c.v. injection of [S9K]TxID effectively attenuated nicotine-induced CPP expression, but considering that [S9K]TxID cannot cross the blood–brain barrier, we propose exploring alternative administration routes for [S9K]TxID in future investigations, such as intranasal administration, which is a noninvasive way of drug delivery that bypasses the blood–brain barrier. In addition, our study suggests that [S9K]TxID may treat nicotine addiction by modulating amino acid metabolism and energy metabolism. Nevertheless, more research is required to validate and examine different metabolites as well as to elucidate the underlying mechanisms. At the same time, it is not clear how the metabolic changes in the hippocampus are related to the effect of the [S9K]TxID on the α3β4 nAChR. In a further exploration, we propose plans to dissect the mechanistic link between [S9K]TxID on the α3β4 nAChR and metabolic changes using α3β4 nAChR knockout mice combined with advanced techniques such as patch-clamp electrophysiology and spatial metabolomics. Therefore, although the results of this study cannot determine the mechanism of action of [S9K]TxID in treating nicotine addiction, they provide a foundation for further research into the therapeutic effects of [S9K]TxID on nicotine addiction.

## 4. Materials and Methods

### 4.1. Animals and Experimental Design

Beijing SPF Biotechnology Co., Ltd. (Beijing, China) provided male C57BL/6J mice, aged 6–8 weeks (SCXK-2019-0010). The housing room was on a 12 h light-dark cycle. Mice were acclimated to handling by the experimenters and the experimental setting prior to the commencement of the experiment.

To assess [S9K] TxID treatment efficacy for nicotine-induced CPP, mice were split into three experimental cohorts for this investigation (*n* = 10 for each group): (1) Saline + Saline with subcutaneous injection (s.c.) of saline and i.c.v. injection of saline; (2) Nicotine + Saline with s.c. injection of nicotine and i.c.v. injection of saline; and (3) Nicotine + [S9K] TxID three nmol with s.c. injection of nicotine and i.c.v. injection of [S9K] TxID. For the open field test and the elevated plus maze test, mice were split into two experimental groups: the saline group and the [S9K]TxID group.

### 4.2. Peptide Synthesis

Linear [S9K]TxID was synthesized by GL Biochem (Shanghai, China) using an Fmoc chemical solid-phase method with side-chain protection and subsequent two-step oxidation to link disulfide bonds. The confirmation of the molecular weight and purity of compounds was achieved through the utilization of LCMS and UPLC techniques [26].

### 4.3. I.c.v. Surgery

Mice were anesthetized with sodium pentobarbital (125 mg/kg, i.p.), the scalp skin was incised, and the bregma was exposed. Unilateral injection sites were prepared by implanting a 2 mm-deep, 26-gauge custom tube into the lateral ventricle (0.6 mm AP, +1.3 mm ML, 2 mm DV relative to Bregma). Mice received an injection volume of 2 µL using an automatic microinjection system (KD Scientific, Holliston, MA, USA) into the lateral ventricle on the day of testing.

### 4.4. Nicotine-Induced Conditioned Place Preference (CPP)

We used two-chambered CPP boxes (15 × 30 × 39 cm). These boxes consisted of two side chambers with different wall and floor patterns to help the mice further distinguish between the two environments, with an auto-monitoring system obtained from RWD, Shenzhen, China. There was a partition between the boxes that separated the two side chambers.

#### 4.4.1. Handling Habituation

Before the experiment, the mice were transported to the CPP behavioral testing room and acclimatized to the room for 2–3 h. The mice were handled daily to acclimatize them to the experimenter. Then the mice were placed in the CPP box with the door allowed free access, connecting the two sides of the box, and allowed to travel freely for 15 min for two days.

#### 4.4.2. Pre-Conditioning Phase

In this phase, the mice were situated within the CPP boxes with the door allowed free access, linking the two side chambers, and a camera recorded their locomotion for 15 min. The time spent by the animals on both sides of the apparatus was analyzed by an automatic animal behavior monitoring system (RWD, Shenzhen, China). The initial preference of the mice was determined.

#### 4.4.3. Conditioning Phase

After the pre-conditioning phase, the mice were conditioned with nicotine/saline for five days, and the connection between the left and right chambers was blocked, confining the mice from traveling freely between the two chambers. Modeling was performed using nicotine (0.5 mg/kg, s.c.). The saline group was administered s.c. injections of saline on both sides of the boxes. The model group and [S9K]TxID group received s.c. injections of nicotine on one side of the chambers and saline on the other. Following the injection, the mice were placed within the designated box and left for 30 min.

#### 4.4.4. Post-Conditioning Phase

In this phase, the mice were situated within the CPP boxes with the door allowed free access, linking the two side chambers, and the camera recorded their locomotion for 15 min. Mice that met the experimental requirements were screened and subjected to lateral ventricle catheterization surgery.

#### 4.4.5. Test Phase

On the test day, mice were treated with [S9K]TxID three nmol or saline 90 min before placing them in the test apparatus, and the camera recorded their locomotion for 15 min. The time spent by the animals on both sides of the apparatus was analyzed by an automatic animal behavior monitoring system.

### 4.5. Open Field Test (OFT)

The mice underwent a 30 min acclimatization period in the behavioral test chamber before the testing. Ninety min. after treating the mice with [S9K]TxID three nmol or saline (i.c.v.), the mice were placed in a test apparatus (40 × 40 × 40 cm; RWD, Shenzhen, China) and recorded for 30 min. After each test, the box was sanitized using 75% ethanol. The recorded video was analyzed, and the data was exported utilizing the Smart 3.0 system (Panlab Harvard Apparatus, Barcelona, Spain).

### 4.6. Elevated Plus Maze Test (ELPM)

The mice underwent a 30 min acclimatization period in the behavioral test chamber prior to testing. The ELPM instrument consists of two open arms and two closed arms (30 × 5 cm each), with the baffles of the closed arms being 15 cm high (RWD, Shenzhen, China). After treating the mice with [S9K]TxID three nmol or saline (i.c.v.) for 90 min, the mice were placed in a test apparatus and recorded for 5 min. After each test, the box was sanitized using 75% ethanol. The recorded video was analyzed, and the data was exported utilizing the Smart 3.0 system (Panlab Harvard Apparatus, Barcelona, Spain).

### 4.7. Hippocampal Untargeted Metabolomics

#### 4.7.1. Preparation of Samples

After the test phase, mice were sacrificed by cervical dislocation, brains were excised, and the hippocampal tissue was dissected. The tissue was rapidly frozen in liquid nitrogen and stored at −80 °C. The following protocol was used to prepare hippocampal samples for metabolomics analysis: Twenty mg of hippocampal tissue was weighed out and added to an 800 μL of solution of methanol/acetonitrile 1:1 volume ratio by volume; the tissue was then ground using a cryo-grinder. Ultrasonicate at 4 °C for 10 min. Allow to stand for 1 h at −20 °C and centrifuge at 12,000 rpm for 15 min. The supernatant was transferred to a new microtube and blow-dried with nitrogen. The sample was re-dissolved in 200 μL of a 1:1 acetonitrile/water solution and then centrifuged. The supernatant was transferred to injection bottles and store at 4 °C for subsequent analysis. QC samples were prepared by mixing 10 μL of supernatant from each hippocampal tissue sample.

#### 4.7.2. Conditions for Mass Spectrometry and Chromatography

Hippocampal metabolomics analyses were executed on a Dionex Ultimate 3000 liquid chromatography system and a Q Exactive Orbitrap mass spectrometry system (Thermo Fisher Scientific, Waltham, MA, USA). The chromatographic column temperature was 40 °C, utilizing a C18 column (1.7 μm, 2.1 mm × 50 mm, Waters, Milford, MA, USA) for the separation process; phase A: aqueous solution with 0.1% formic acid; phase B: acetonitrile; flow rate: 0.3 mL/min; injection volume: 2 μL. The gradient was as follows: 0–2 min, 95% A; 2–13 min, 95–0% A; 13–16 min, 0% A; 16–16.1 min, 0–95% A; 16.1–18 min, 95%. The mass spectrometric conditions were as follows: electrospray voltage, 3000 V; auxiliary gas heater temperature, 420 °C; mass range, 100–1200 m/z.

#### 4.7.3. Data Processing

The multivariate statistical analysis was conducted utilizing PCA and OPLS-DA. Differential metabolites were identified by selecting metabolites with VIP > 1 and *p* < 0.05. The identification of these metabolites was verified through the HMDB database and PubChem. Moreover, the MetaboAnalyst 6.0 was utilized to analyze related metabolic pathways.

### 4.8. Statistical Analysis

Statistical analysis was conducted utilizing GraphPad Prism 8 and all results were reported as mean ± SEM. The CPP scores were determined by calculating the difference between the initial duration spent in the drug-paired chamber and the duration spent in that chamber during the following trial. One-way ANOVA was applied to compare means among groups. All differences between groups were considered statistically significant at *p* < 0.05. All quantitative results in vitro were obtained from at least 3 samples, while animal studies consisted of 8–10 samples.

## 5. Conclusions

Treating nicotine addiction has long been a major concern. Here, we demonstrated that [S9K]TxID effectively attenuated nicotinic-induced CPP expression, while it had no inhibitory effect on the central nervous system, as determined through pharmacodynamic experiments. The metabolomics analysis indicated that [S9K]TxID modulated the dysregulation of various metabolites in the hippocampus of mice with nicotine addiction. Among these metabolites, phenylalanine was the main differential metabolite following [S9K]TxID treatment, and [S9K]TxID effectively reversed the elevated hippocampal phenylalanine levels. [S9K]TxID exerts anti-nicotine addiction effects by ameliorating the disorders of amino acid and energy metabolic pathways to restore nicotine-addicted mice to normal levels. In conclusion, the combined analysis of pharmacodynamic and metabolomic data provides a basis for further investigation on the potential mechanism of action of [S9K]TxID in nicotine addiction, positioning [S9K]TxID as a potential candidate for development as a smoking cessation medication.

## Figures and Tables

**Table 1:** Time spent in drug-paired compartments prior to the administration of antagonist [S9K]TxID in mice with nicotine-induced conditioned place preference (CPP) (s).

Group		Pre-Condition	Post-Condition	CPP Score
Saline	Saline	304.3 ± 22.4	333.6 ± 27.1	29.3 ± 18.7
Nicotine	Saline	273.6 ± 15.2	431.2 ± 21.2 **	157.6 ± 17.1 ***
	[S9K]TxID 3 nmol	304.5 ± 16.7	448.2 ± 12.1 **	143.7 ± 19.8 ***

Data are the mean ± SEM of 10 mice per group. CPP score (s) = the time spent in drug-paired chamber post-conditioning minus pre-conditioning. ** *p* < 0.01, *** *p* < 0.001 compared with Saline + Saline group.

**Table 2:** Time spent in drug-paired compartments after the administration of antagonist [S9K]TxID in mice with nicotine-induced CPP (s).

Group		Pre-Condition	Test	CPP Score
Saline	Saline	304.3 ± 22.4	264.5 ± 41.8	−39.8 ± 25.3
Nicotine	Saline	273.6 ± 15.2	387.2 ± 24.9 *	113.6 ± 23.5 **
	[S9K]TxID 3 nmol	304.5 ± 16.7	301.7 ± 26.2	−2.8 ± 36.2 ^#^

Data are the mean ± SEM of 10 mice per group. CPP score (s) = the time spent in drug-paired chamber test minus pre-conditioning. * *p* < 0.05, ** *p* < 0.01 compared with Saline + Saline group; ^#^ *p* < 0.05 compared with the Nicotine + Saline group.

**Table 3:** Differential metabolites in the hippocampus after [S9K]TxID treatment.

No.	Metabolites	Adduct	Retention Time (min)	VIP	*p* Value	Trend ([S9K]TxID/Control)	Trend (Model/Control)	Trend ([S9K]TxID/Model)
1	4-Dodecylbenzenesulfonic acid	[M-H]-	17.05	2.18	1.5 × 10^−3^	↑	↑	—
2	Stearic acid	[M-H]-	15.14	2.07	2.91 × 10^−6^	↑	↑	↓
3	4-Oxoproline	[M-H]-	0.51	1.53	4.27 × 10^−4^	↑	↑	—
4	D-Malic acid	[M-H]-	0.53	4.22	1.35 × 10^−5^	↑	↑	—
5	N-Acetylaspartylglutamic acid	[M-H]-	0.72	1.51	2.07 × 10^−2^	↑	↑	—
6	D-Ribose 5-phosphate	[M-H]-	0.50	1.30	2.34 × 10^−5^	↑	↑	—
7	Ascorbic acid	[M-H]-	0.51	6.69	1.05 × 10^−3^	↑	↑	—
8	Glutamic acid	[M-H]-	0.49	2.98	1.25 × 10^−3^	↑	↑	—
9	Phenylalanine	[M-H]-	10.03	1.49	1.51 × 10^−3^	—	↑	↓
10	Uridine 5′-diphosphogalactose	[M-H]-	0.57	1.78	6.49 × 10^−8^	↑	↑	—
11	D-Glutamine	[M-H]-	0.46	2.04	3.17 × 10^−3^	↑	↑	—
12	ADP	[M-H]-	0.73	1.06	6.02 × 10^−3^	↑	↑	—
13	Palmitoylcarnitine	[M + H]+	10.62	3.83	6.26 × 10^−4^	↑	↑	—
14	Stearamide	[M + H]+	13.72	2.19	3.19 × 10^−3^	↑	↑	—
15	L-Norleucine	[M + H]+	0.80	1.72	4.37 × 10^−2^	—	↓	↑
16	Cholecalciferol	[M + H]+	13.33	2.32	4.88 × 10^−3^	↑	↑	—
17	Docosanamide	[M + H]+	15.54	1.60	1.62 × 10^−2^	—	↑	↓
18	N-Arachidonoyl Taurine	[M-H]-	15.01	1.09	2.16 × 10^−3^	—	—	↓
19	Nicotinamide	[M + H]+	0.54	4.66	1.82 × 10^−3^	—	—	↑
20	Taurine	[M + H]+	0.53	1.47	1.97 × 10^−2^	—	—	↑
21	Oxidized glutathione	[M + H]+	0.55	2.38	1.48 × 10^−3^	↑	—	↑

Note: VIP: variable importance in projection; ↑: up-regulated, ↓: down-regulated.

## Data Availability

The data presented in this study are available on request from the corresponding authors.

## Supplementary Materials

The following supporting information can be downloaded at: https://www.mdpi.com/article/10.3390/md24010043/s1, Figure S1. The typical total ion chromatograms (TICs); Figure S2. The typical total ion chromatograms (TICs); Figure S3. (A) Three-dimensional PCA diagram for each group in the positive ion modes. (B) Three-dimensional PCA diagram for each group in negative ion modes; Table S1. Information of identified compounds in metabolomics; Table S2. Differential metabolites in the hippocampus after [S9K]TxID treatment.

## Abbreviations

The following abbreviations are used in this manuscript:

nAChRs	nicotine acetylcholine receptors
CPP	conditioned place preference
NRT	nicotine replacement therapy
MHb	medial habenula
IPN	interpeduncular nucleus
18-MC	18-methoxycoronidine
i.c.v.	intracerebroventricular injections
s.c.	subcutaneous injection
OFT	open field test
ELPM	elevated plus maze test
PCA	principal component analysis
OPLS-DA	orthogonal partial least-squares discriminant analysis
QC	quality control
VIP	variable important in projection
KEGG	kyoto encyclopedia of genes and genomes
NAcc	nucleus accumbens
NAD^+^	nicotinamide adenine dinucleotide
NADP^+^	nicotinamide adenine dinucleotide phosphate
TCA	tricarboxylic acid
WHO	World Health Organization
CNS	central nervous system
