# Supplementary material for: Hippocampal Metabolomics Reveal the Mechanism of α-Conotoxin [S9K]TxID Attenuating Nicotine Addiction
Source: Mar Drugs. 2026 Jan 15;24(1):43. doi: 10.3390/md24010043 (PMC12842921; doi:10.3390/md24010043)
Supplement: Supplementary file 1 [file marinedrugs-24-00043-s001.zip › marinedrugs-3999420-supplementary.pdf]

Supplementary Material

Hippocampal metabolomics reveal the mechanism of α-conotoxin [S9K]TxID attenuating nicotine addiction

Meiting Wang [1], Weifeng Xu [1], Huanbai Wang [1], Cheng Cui [1], Rongyan He [1], Xiaodan Li [2], Jinpeng Yu [1], J. Michael McIntosh [3,4], Dongting Zhangsun [1,2,*] and Sulan Luo [1,2,*]

1 Guangxi Key Laboratory of Special Biomedicine, School of Medicine, College of Life Science and Technology, Guangxi University, Nanning 530004, China.

2 Key Laboratory of Tropical Biological Resources of Ministry of Education, Hainan University, Haikou 570228, China.

3 Departments of Biology and Psychiatry, University of Utah, Salt Lake City, UT 84112, USA.

4 George E. Wahlen Veterans Affairs Medical Center, Salt Lake City, UT 84108, USA.

*Correspondence: zhangsundt@163.com (D.Z.); sulan2021@gxu.edu.cn (S.L.)

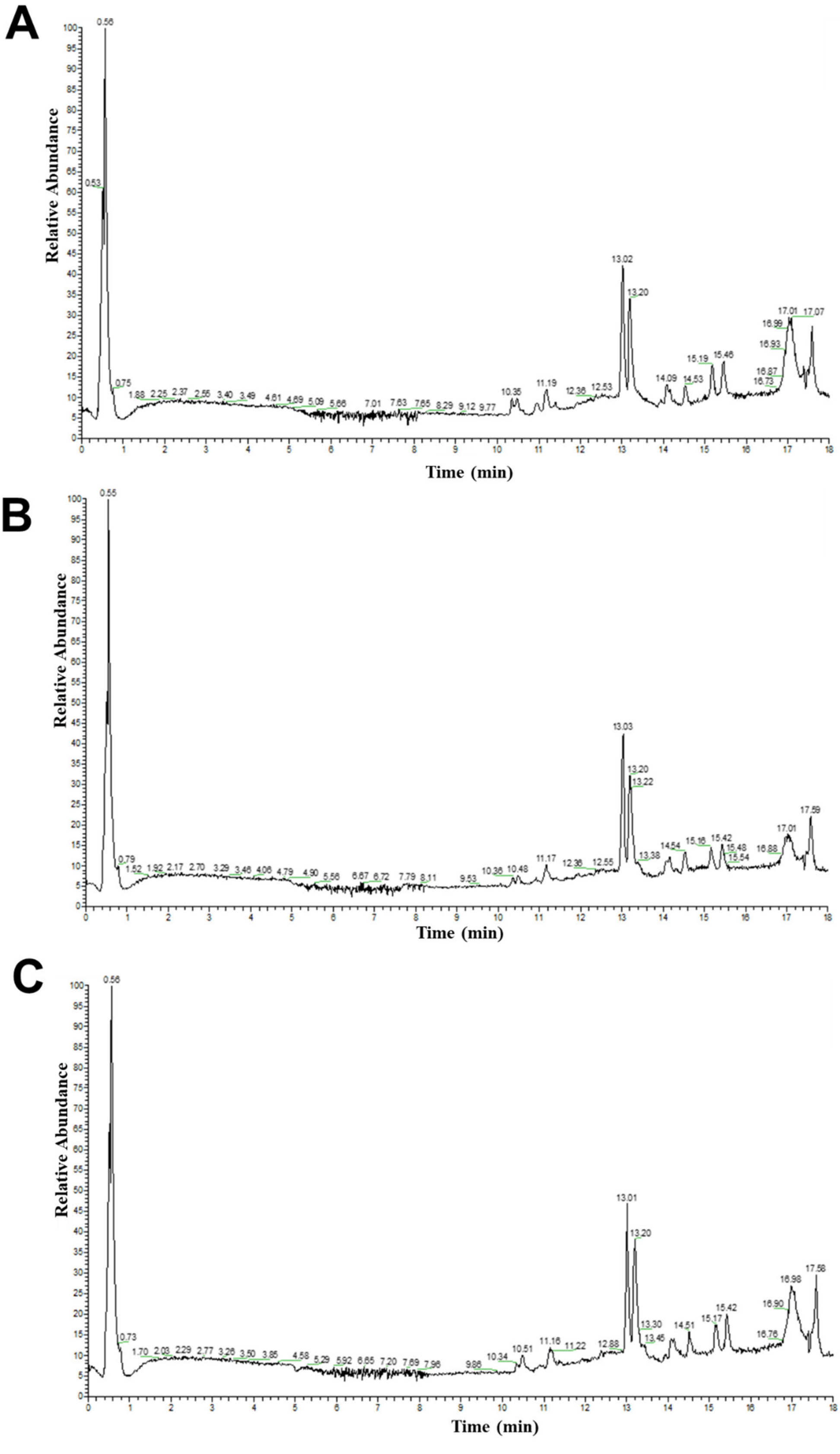

Figure S1. The typical total ion chromatograms (TICs). (A) The TIC of the Control group obtained from the ESI negative. (B) The TIC of the Model group obtained from

the ESI negative. (C) The TIC of the [S9K]TxID group obtained from the ESI negative.

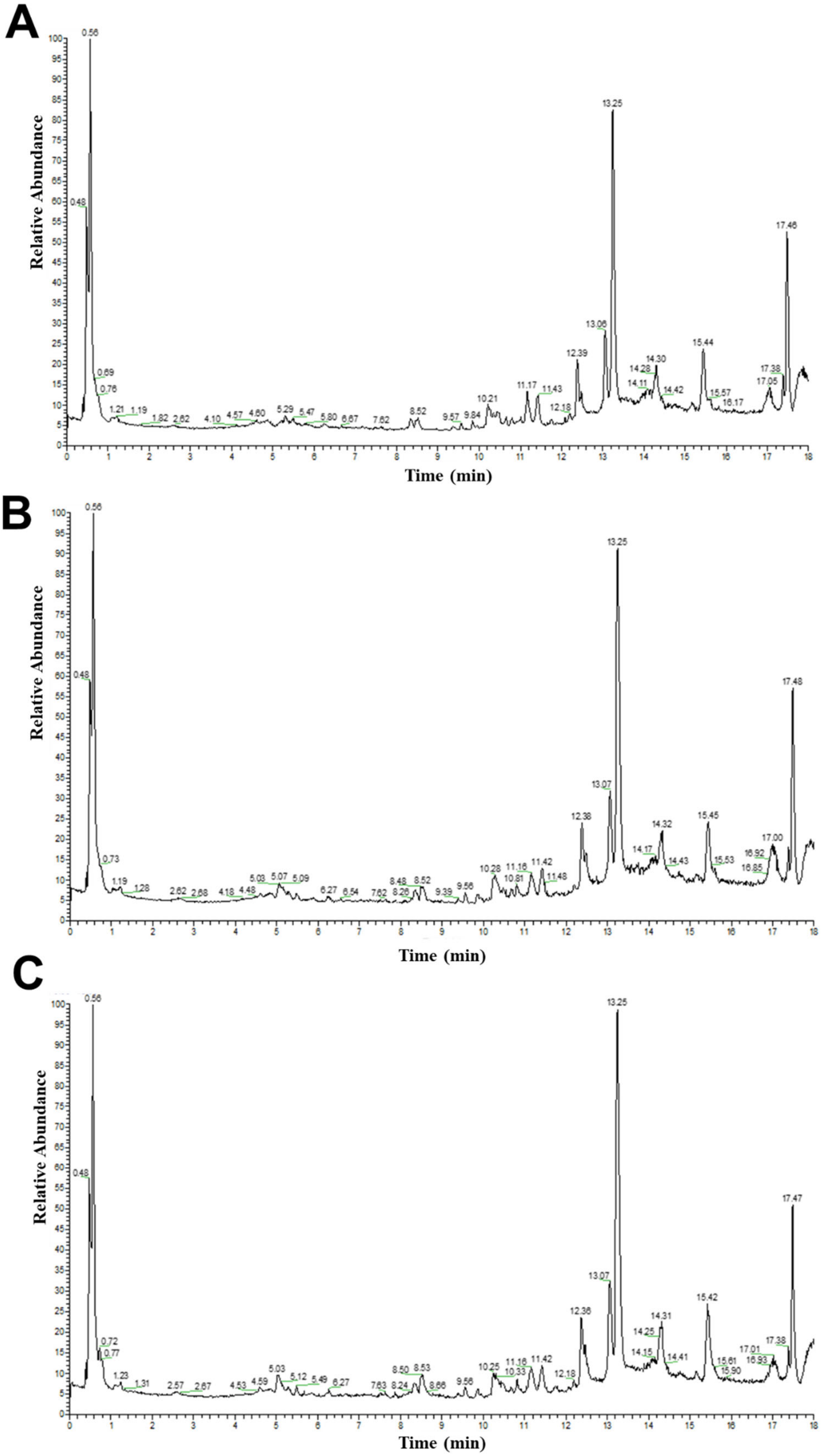

Figure S2. The typical total ion chromatograms (TICs). (A) The TIC of the Control

group obtained from the ESI positive. (B) The TIC of the Model group obtained from the ESI positive. (C) The TIC of the [S9K]TxID group obtained from the ESI positive.

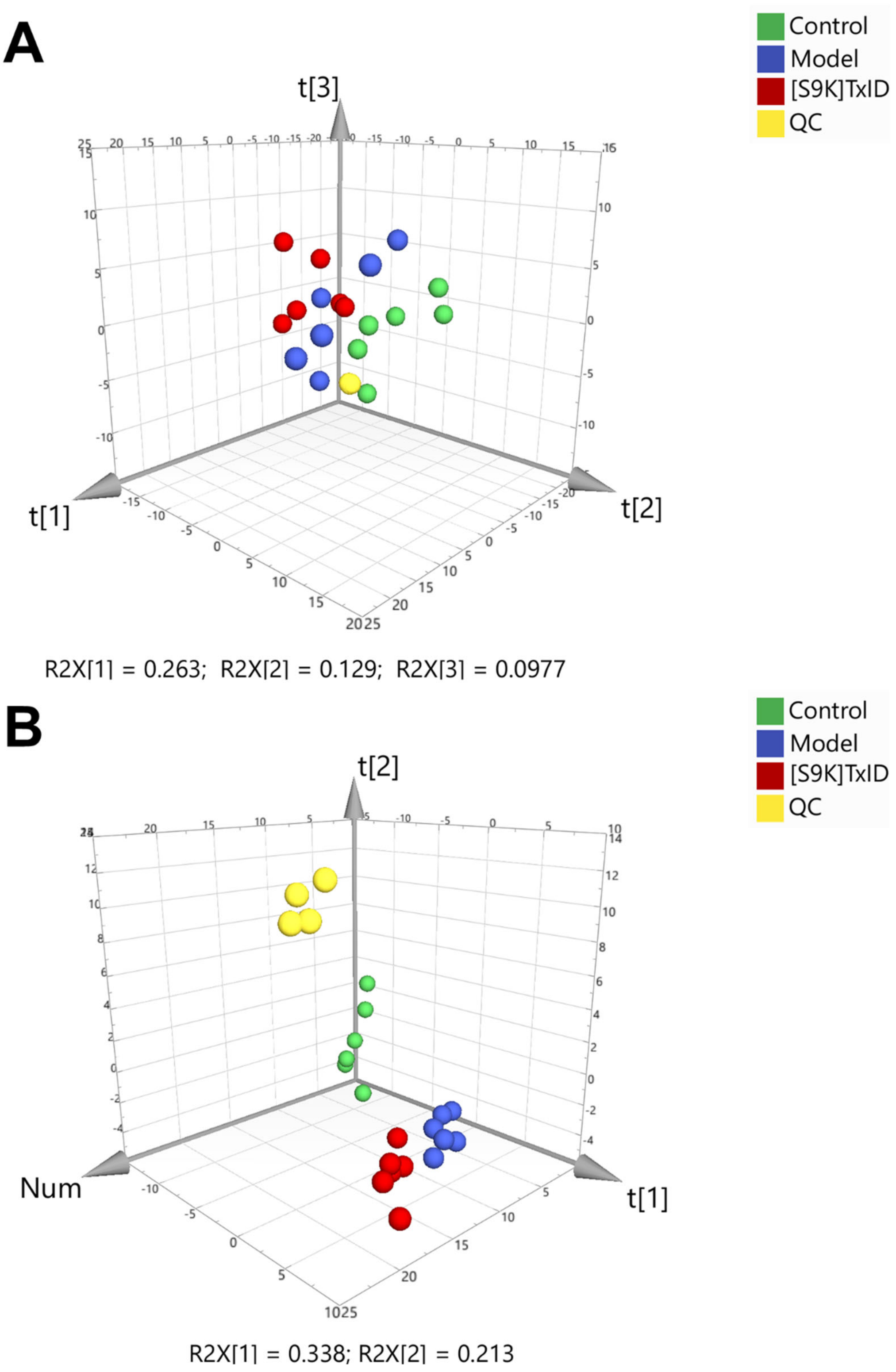

Figure S3. (A) Three-dimensional PCA diagram for each group in the positive ion modes. R2X[1]: Principal Component 1 (26.3% variance explained); R2X[2]: Principal

Component 2 (12.9% variance explained); R2[3]: Principal Component 3 (9.8% variance explained). (B) Three-dimensional PCA diagram for each group in negative ion modes. R2X[1]: Principal Component 1 (33.8% variance explained); R2X[2]: Principal Component 2 (21.3% variance explained). In the three-dimensional PCA diagram, yellow points represent the QC samples, green points represent the Control group, blue points represent the Model group, and red points represent the [S9K]TxID group.

Table S1. Information of identified compounds in metabolomics

Number	Compound name	Formula	RT (min)	Detected m/z	Adduct
1	4-Dodecylbenzenesulfonic acid	C18 H30 O3 S	17.05	325.1828	[M-H]-
2	Dihomo-gamma-linolenic acid	C20 H34 O2	13.64	305.2474	[M-H]-
3	11-HETE	C20 H32 O3	11.21	319.2264	[M-H]-
4	Xanthine	C5 H4 N4 O2	0.53	151.0244	[M-H]-
5	Stearic acid	C18 H36 O2	15.14	283.2630	[M-H]-
6	Dihomo-alpha-linolenic acid	C20 H34 O2	13.88	305.2474	[M-H]-
7	Ostruthin	C19 H22 O3	12.37	297.1518	[M-H]-
8	16-Hydroxyhexadecanoic acid	C16 H32 O3	12.90	271.2268	[M-H]-
9	4-Oxoproline	C5 H7 N O3	0.51	128.0335	[M-H]-
10	Indole-3-acetyl-L-aspartic acid	C14 H14 N2 O5	9.79	289.0859	[M-H]-
11	2-Mercaptobenzothiazole	C7 H5 N S2	6.94	165.9774	[M-H]-
12	Lignoceric acid	C24 H48 O2	14.40	367.3566	[M-H]-
13	Arachidonic acid	C20 H32 O2	13.84	303.2317	[M-H]-
14	N-Arachidonoyl taurine	C22 H37 N O4 S	15.01	410.2352	[M-H]-
15	Oleic Acid	C18 H34 O2	14.51	281.2474	[M-H]-

16	β-D-Fructose 6-phosphate	C6 H13 O9 P	0.50	259.0213	[M-H]-
17	Vicenin 2	C27 H30 O15	11.38	593.1585	[M-H]-
18	Malic acid	C4 H6 O5	0.49	133.0125	[M-H]-
19	D-Malic acid	C4 H6 O5	0.53	133.0125	[M-H]-
20	N-Acetylaspartylglutamic acid	C11 H16 N2 O8	0.72	303.0822	[M-H]-
21	D-Ribose 5-phosphate	C5 H11 O8 P	0.50	229.0104	[M-H]-
22	N-Arachidonoyl Dopamine	C28 H41 N O3	11.26	438.2980	[M-H]-
23	Uridine diphosphate glucuronic acid	C15 H22 N2 O18 P2	0.63	579.0248	[M-H]-
24	Guanosine	C10 H13 N5 O5	0.76	282.0832	[M-H]-
25	Adenine	C5 H5 N5	0.71	134.0454	[M-H]-
26	Ascorbic acid	C6 H8 O6	0.51	175.0232	[M-H]-
27	Benzothiazolone	C7 H5 N O S	6.46	150.0002	[M-H]-
28	Glutamic acid	C5 H9 N O4	0.49	146.0442	[M-H]-
29	D-2-Hydroxyglutaric	C5 H8 O5	0.49	147.0281	[M-H]-
30	Phenylalanine	C9 H11 N O2	10.03	164.0700	[M-H]-
31	Uridine 5'-diphosphogalactose	C15 H24 N2 O17 P2	0.57	565.0452	[M-H]-
32	Docosapentaenoic acid (22n-6)	C22 H34 O2	13.57	329.2474	[M-H]-

33	D-Glutamine	C5 H10 N2 O3	0.46	145.0603	[M-H]-
34	Flavin adenine dinucleotide (FAD)	C27 H33 N9 O15 P2	5.02	784.1476	[M-H]-
35	S-Lactoylglutathione	C13 H21 N3 O8 S	0.79	378.0962	[M-H]-
36	Adenosine diphosphate (ADP)	C10 H15 N5 O10 P2	0.73	426.0206	[M-H]-
37	Creatine	C4 H9 N3 O2	0.48	132.0761	[M+H]+
38	Adenosine	C10 H13 N5 O4	0.56	268.1023	[M+H]+
39	Nicotinamide	C6 H6 N2 O	0.54	123.0547	[M+H]+
40	3'-Adenosine monophosphate	C10 H14 N5 O7 P	0.51	348.0680	[M+H]+
41	5'-Methylthioadenosine	C11 H15 N5 O3 S	2.61	298.0949	[M+H]+
42	Palmitoylethanolamide	C18 H37 N O2	12.55	300.2876	[M+H]+
43	L-Carnitine	C7 H15 N O3	0.47	162.1115	[M+H]+
44	Palmitoylcarnitine	C23 H45 N O4	10.62	400.3395	[M+H]+
45	Hexanoylcarnitine	C13 H25 N O4	5.54	260.1841	[M+H]+
46	L-Acetylcarnitine	C9 H17 N O4	0.54	204.1218	[M+H]+
47	Taurine	C2 H7 N O3 S	0.53	126.0214	[M+H]+
48	gamma-Glutamylglutamic acid	C10 H16 N2 O7	0.52	277.1012	[M+H]+
49	D-Tryptophan	C11 H12 N2 O2	2.40	205.0959	[M+H]+

50	L-Threonine	C4 H9 N O3	0.48	120.0651	[M+H]+
51	DL-Glutamine	C5 H10 N2 O3	0.49	147.0756	[M+H]+
52	N6,N6,N6-Trimethyl-L-lysine	C9 H20 N2 O2	0.42	189.1587	[M+H]+
53	Indoleacrylic acid	C11 H9 N O2	2.59	188.0694	[M+H]+
54	DL-Arginine	C6 H14 N4 O2	0.41	175.1180	[M+H]+
55	L-Histidine	C6 H9 N3 O2	0.47	156.0758	[M+H]+
56	Nobiletin	C21 H22 O8	8.52	403.1360	[M+H]+
57	Stearamide	C18 H37 N O	13.72	284.2927	[M+H]+
58	Tris(2-ethylhexyl) phosphate	C24 H51 O4 P	16.66	435.3568	[M+H]+
59	2-Amino-1,3,4-octadecanetriol	C18 H39 N O3	8.32	318.2980	[M+H]+
60	Linoleoyl Ethanolamide	C20 H37 N O2	11.93	324.2876	[M+H]+
61	Tangeritin	C20 H20 O7	9.03	373.1258	[M+H]+
62	Methionine sulfoxide	C5 H11 N O3 S	0.59	148.0418	[M+H-H2O]+
63	Propionylcarnitine	C10 H19 N O4	0.85	218.1374	[M+H]+
64	Oxidized glutathione	C20 H32 N6 O12 S2	0.55	613.1550	[M+H]+
65	Pentaethylene glycol	C10 H22 O6	2.24	239.1473	[M+H]+
66	Oleamide	C18 H35 N O	10.85	282.2772	[M+H]+

67	L-Norleucine	C6 H13 N O2	0.80	132.1011	[M+H]+
68	Nipecotic acid	C6 H11 N O2	0.40	147.1120	[M+NH4]+
69	Cholecalciferol	C27 H44 O	13.33	385.3435	[M+H]+
70	1-Linoleoyl glycerol	C21 H38 O4	13.40	355.2820	[M+H]+
71	Corticosterone	C21 H30 O4	7.62	347.2193	[M+H]+
72	5-Hydroxyindoleacetic acid	C10 H9 N O3	3.92	192.0643	[M+H]+
73	Docosanamide	C22 H45 N O	15.54	340.3548	[M+H]+
74	Monoolein	C21 H40 O4	13.38	339.2870	[M+H-H2O]+
75	Arachidonoyl amide	C20 H33 N O	12.42	304.2610	[M+H]+
76	Leucocrystal violet	C25 H31 N3	10.62	374.2641	[M+H]+
77	Cuminaldehyde	C10 H12 O	12.48	149.0951	[M+H]+
78	17(18)-EpETE	C20 H30 O3	7.78	319.2247	[M+H]+
79	Arachidonic acid methyl ester	C21 H34 O2	14.43	319.2610	[M+H]+
80	2-Arachidonoyl glycerol	C23 H38 O4	12.46	379.2817	[M+H]+
81	Testosterone acetate	C21 H30 O3	8.57	331.2241	[M+H]+
82	Prolylleucine	C11 H20 N2 O3	2.04	229.1532	[M+H]+
83	2,4-Dimethylbenzaldehyde	C9 H10 O	11.90	135.0796	[M+H]+

84	12-Aminododecanoic acid	C12 H25 N O2	7.16	216.1945	[M+H]+
85	Alpha-Linolenoyl ethanolamide	C20 H35 N O2	12.42	322.2717	[M+H]+
86	Pinolenic acid	C18 H30 O2	8.19	296.2564	[M+NH4]+
87	(-)-Caryophyllene oxide	C15 H24 O	12.39	238.2147	[M+NH4]+
88	Ureidopropionic acid	C4 H8 N2 O3	0.59	265.1105	[2M+H]+

Table S2. Differential metabolites in the hippocampus after [S9K]TxID treatment.

No.	Metabolites	Adduct	Fold change ([S9K]TxID /Control)	Fold change (Model /Control)	Fold change ([S9K]TxID /Model)
1	4-Dodecylbenzenesulfonic acid	[M-H]-	2.31	1.75	—
2	Stearic acid	[M-H]-	6.21	8.32	0.75
3	4-Oxoproline	[M-H]-	3.82	3.46	—
4	D-Malic acid	[M-H]-	2.55	2.58	—
5	N-Acetylaspartylglutamic acid	[M-H]-	3.07	2.50	—
6	D-Ribose 5-phosphate	[M-H]-	1.80	1.65	—
7	Ascorbic acid	[M-H]-	1.94	1.76	—

8	Glutamic acid	[M-H]-	1.47	1.41	—
9	Phenylalanine	[M-H]-	—	1570.87	0.06
10	Uridine 5'-diphosphogalactose	[M-H]-	3.32	3.09	—
11	D-Glutamine	[M-H]-	1.57	1.71	—
12	ADP	[M-H]-	1.68	1.52	—
13	Palmitoylcarnitine	[M+H]+	1.35	1.45	—
14	Stearamide	[M+H]+	1.45	1.83	—
15	L-Norleucine	[M+H]+	—	0.82	1.27
16	Cholecalciferol	[M+H]+	1.32	1.48	—
17	Docosanamide	[M+H]+	—	2.65	0.49
18	N-Arachidonoyl Taurine	[M-H]-	—	—	0.48
19	Nicotinamide	[M+H]+	—	—	1.21
20	Taurine	[M+H]+	—	—	1.45
21	Oxidized glutathione	[M+H]+	1.43	—	1.43
